# Enhanced Bio-BTX Formation by Catalytic Pyrolysis of Glycerol with *In Situ* Produced Toluene as the Cofeed

**DOI:** 10.1021/acssuschemeng.4c00451

**Published:** 2024-03-25

**Authors:** Fukang Wang, Thomas Sjouke Kramer, Bin Yan, Lin Zhu, Yuezhao Zhu, Andre Heeres, Diana Ciolca, Hero Jan Heeres, Songbo He

**Affiliations:** † Joint International Research Laboratory of Circular Carbon, Nanjing Tech University, 211816 Nanjing, P. R. China; ‡ Green Chemical Reaction Engineering, Engineering and Technology Institute Groningen, University of Groningen, 9747 AG Groningen, The Netherlands; § Hanze University of Applied Sciences, 9747 AS Groningen, The Netherlands; ∥ BioBTX BV, 9747 AA Groningen, The Netherlands

**Keywords:** refinery coprocessing, bioaromatics, ZSM-5, synergy effect, sustainable catalysis performance

## Abstract

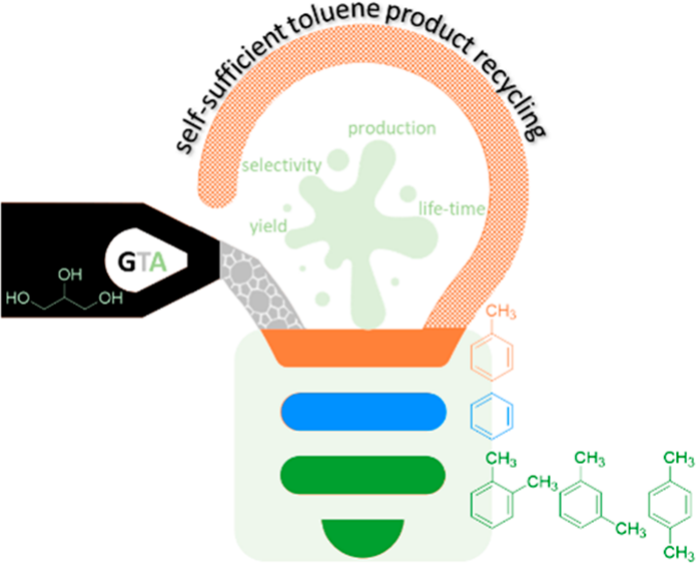

The catalytic coconversion of glycerol and toluene (93/7 wt %) over a technical H-ZSM-5/Al_2_O_3_ (60–40 wt %) catalyst was studied, aiming for enhanced production of biobased benzene, toluene, and xylenes (bio-BTX). When using glycerol/toluene cofeed with a mass ratio of 93/7 wt %, a peak BTX carbon yield of 29.7 ± 1.1 C.% (at time on stream (TOS) of 1.5–2.5 h), and an overall BTX carbon yield of 28.7 C.% (during TOS of 8.5 h) were obtained, which are considerably higher than those (19.1 ± 0.4 C.% and 11.0 C.%) for glycerol alone. Synergetic effects when cofeeding toluene on the peak and overall BTX carbon yields were observed and quantified, showing a relative increase of 3.1% and 30.0% for the peak and overall BTX carbon yield (based on the feedstock). These findings indicate that the strategy of cofeeding *in situ* produced toluene for the conversion of glycerol to aromatics has potential to increase BTX yields. In addition, BTX production on the catalyst (based on the fresh catalyst during the first run for TOS of 8.5 h and without regeneration) is significantly improved to 0.547 ton ton^–1^_catalyst_ (excluding the 76% of toluene product that is 0.595 ton ton^–1^_catalyst_ for the recycle in the cofeed) for glycerol/toluene cofeed, which was 0.426 ton ton^–1^_catalyst_ for glycerol alone. In particular, this self-sufficient toluene product recycling strategy is advantageous for the production and selectivity (relative increase of 84.4% and 43.5% during TOS of 8.5 h) of biobased xylenes.

## Abstract 2

An efficient cofeeding strategy with the use of *in situ* produced toluene for the catalytic conversion of glycerol to biobased BTX is provided.

## Introduction

Monocyclic aromatics such as benzene, toluene, and xylenes (BTX) are basic building blocks for the production of various consumer products (*e.g*., plastics, cosmetics, and adhesives) *via* alkylation, acylation, carboxylation, reduction, oxidation, nitration, amination, sulfonation, and halogenation reactions.^1^ The global BTX market was 129 million tons in 2022 and is projected to grow to 180 million tons by 2031.^2^ Currently, BTX is mainly produced from fossil sources, namely, reformate (*ca*. 68%, from catalytic reforming), pyrolysis gasoline (*ca*. 29%, from steam cracking), and light oil (*ca*. 3%, from coke oven),^3^ the use of which has major drawbacks such as CO_2_ emissions and BTX price volatility (Figure S1).

Recently, the concepts of biobased and circular economies^4^ have been proposed to tackle climate change and to address sustainability challenges. This trend is expected to change the BTX market by increased demands for bio-BTX, *e.g*., for the production of bioplastics (such as biobased PET and PS, Figure S2).^5^ For the latter, the global annual production is projected to grow from *ca*. 2.2 M tons in 2022 to *ca*. 6.3 million tons in 2027.^6^

Defossilization of chemical production requires innovative technologies.^7^ Catalytic pyrolysis is an emerging platform technology, which is versatile, scalable, and capable of the direct conversion of biomass to biobased chemicals and/or biofuels.^8^ Various biobased feedstocks, such as pinewood sawdust,^9^ lignin,^10^ microalgae,^11^ paper sludge,^12^ black liquor,^13^ waste cooking oil,^14^ Jatropha residues,^15^ bioethanol,^16^ and free fatty acids,^17^ have been studied for the synthesis of bio-BTX using, *e.g*., ZSM-5-based catalysts.^18^

Glycerol is one of the most abundant nonedible biomass sources and is considered a green platform chemical to synthesize various commodity chemicals. Examples are gasification to syngas and dehydration to acrolein^19^ and also the catalytic pyrolysis to BTX.^20^ Glycerol is partially (*ca*. 1/3) produced in the soap or fatty acid industry and is a byproduct from the biodiesel industry.^21^ The latter glycerol is often contaminated with salts, fatty acids, and alcohols and is known as crude glycerol. The estimated production of crude glycerol is projected to be about 4 million tonnes by 2024.^22^

The catalytic pyrolysis of crude glycerol to bio-BTX has been investigated. A first lab-scale demonstration was accomplished by the authors in 2016 using a continuous fixed-bed reactor with a crude glycerol feed rate of 200 g h^–1^, giving a total bio-BTX yield of 8.1 wt % (on weight basis, equivalent to 14.6 C.% on carbon basis) over a shaped H-ZSM-5/bentonite catalyst.^23^ A pilot-scale Integrated Cascading Catalytic Pyrolysis (ICCP) process^24^ has been operated by BioBTX B.V., The Netherlands, since 2018 using a continuous fluidized-bed reactor with a crude glycerol feed capacity of 100 kg h^–1^.^25^

A major issue for the catalytic pyrolysis of glycerol is reversible catalyst deactivation related to coke formation, as well as irreversible catalyst deactivation after a few reaction-regeneration cycles.^23^ A benchmark study using pure glycerol and unmodified H-ZSM-5 zeolite^26^ revealed that irreversible catalyst deactivation is related to dealumination of the H-ZSM-5 framework,^26,27^ which could be moderated by using a proper binder, *e.g*., Al_2_O_3_, to formulate an H-ZSM-5/Al_2_O_3_ (60/40, wt %) catalyst.^28,29^

Nevertheless, there is an incentive to increase the state-of-the-art catalyst performance for glycerol to aromatics (GTA) to enhance the chance of success for industrial implementation. Considerable efforts have been made on tailoring the properties of the H-ZSM-5 zeolite (*e.g*., Lewis/Brønsted acidity, microporosity, crystallinity, and hydrophilicity) by modification with various metals (such as Zn,^30^ Ga,^31^ and Sn^32^) and by using zeolites with hierarchical structures (*e.g*., *via* alkali treatment^33^ or using templates^34^).

**Figure 1:**
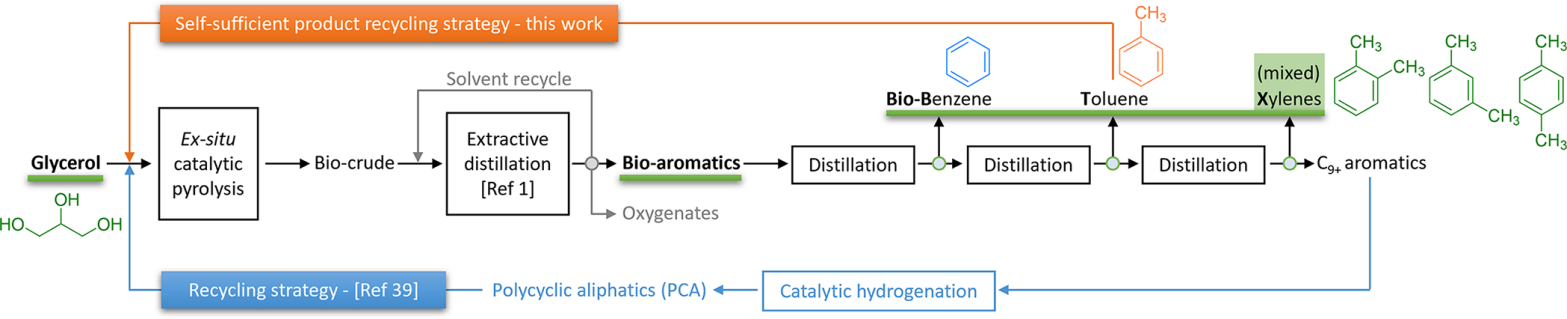
Product recycling strategy for improved production of bio-BTX from glycerol conversion to aromatics (GTA).

Alternatively, increasing the hydrogen-to-carbon effective ratio (H/C_eff_) of the feed by cofeeding glycerol (of which the H/C_eff_ is 0.67) with various cofeeds with a higher H/C_eff_ such as alcohols,^35,36^ alkanes,^36,37^ and free fatty acids,^36,38^ has also been explored. Synergistic effects of the cofeeds on peak BTX carbon yield, BTX productivity (per cycle of reaction-regeneration), catalyst lifetime, and catalyst regenerability have been observed, and a cofeeding strategy has been proposed.^36,38^ Of particular interest is the observation that polycyclic aliphatics (PCAs) may also be used as the cofeed.^39^ These PCAs can be obtained from the partial hydrogenation of polycyclic aromatic hydrocarbons (PAHs),^24,39^ which are coproduced during catalytic pyrolysis of glycerol to BTX.^23^ Modeling studies showed that the overall BTX yield may in theory be increased from 10 wt % for once-through operation to up to *ca*. 16 wt % after recycling the PCA.^39^ This product recycling strategy (Figure 1) shows that the product (though after a catalytic conversion step) is an attractive cofeed for the cofeeding strategy to meet catalysis performance metrics^40^ for GTA.

Triggered by the results above, the authors here report studies on the use of one of the BTX products (namely, toluene) without postcatalytic conversion as the cofeed for GTA (Figure 1). Toluene can be separated by extractive distillation^1^ from the other aromatics (Figure 1). The toluene fraction has the lowest value, and its disproportionation to benzene and *p*-xylene is often used on a commercial scale to increase revenues. In this work, the catalytic coconversion of glycerol and toluene (93/7 wt %) was investigated, and the synergistic effects of the cofeeding on the peak and overall carbon yields for BTX were quantified.

## Experimental Section

Catalytic pyrolysis of individual feed (namely, glycerol and toluene, both analytical grade) and copyrolysis of cofeeds (namely, glycerol/toluene, 93/7 wt %) were performed on a fixed bed reactor (with two feeding lines and optimized in previous work)^36,38^ using 10 g of a technical H-ZSM-5/Al_2_O_3_ (60/40 wt %) catalyst (optimized in previous work)^28,29^ for a time on stream (TOS) of 12 h. Other reaction parameters are reaction temperature of 550 °C, atmospheric pressure, weight hourly space-velocity (WHSV) of the (co)feeds of 1 h^–1^, and N_2_ flow of 50 mL min^–1^. These parameters were optimized in previous work^29^ and have been used to study catalytic copyrolysis of glycerol with cofeeds (such as fatty acids, alcohols, and alkanes).^36,38^ Extended experimental procedures and product analysis are included in the Supporting Information. It needs to be noted here that the cofeed ratio of glycerol/toluene (93/7 wt %) in this preliminary work is only an example and can be further optimized.

## Results and Discussion

### Catalytic Conversion of Glycerol

**Figure 2:**
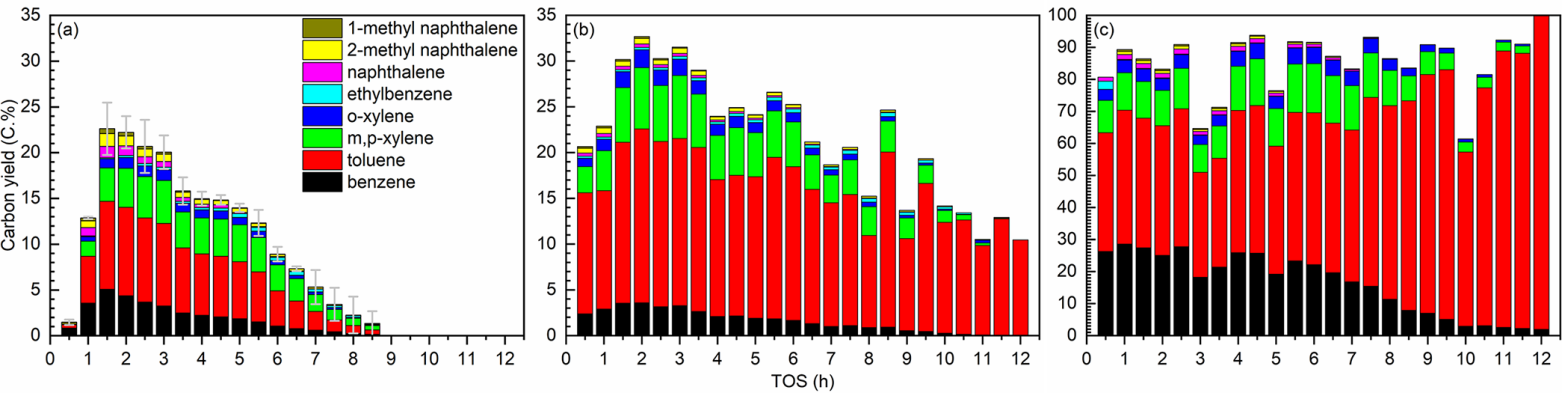
Carbon yields of aromatics vs TOS for catalytic conversion of individual glycerol (a) and toluene (c), and catalytic coconversion of glycerol and toluene (93/7 wt %, b). Reaction conditions: H-ZSM-5/Al_2_O_3_ (60/40 wt %) catalyst of 10 g, WHSV of the (co)feeds of 1 h^–1^, N_2_ flow of 50 mL min^–1^, reactor temperature of 550 °C, and atmospheric pressure. (a) Data are adapted with permission under a Creative Commons CC-BY 4.0 DEED from ref (29) Copyright 2021 The Authors. Published by Elsevier B.V.

**Figure 3:**
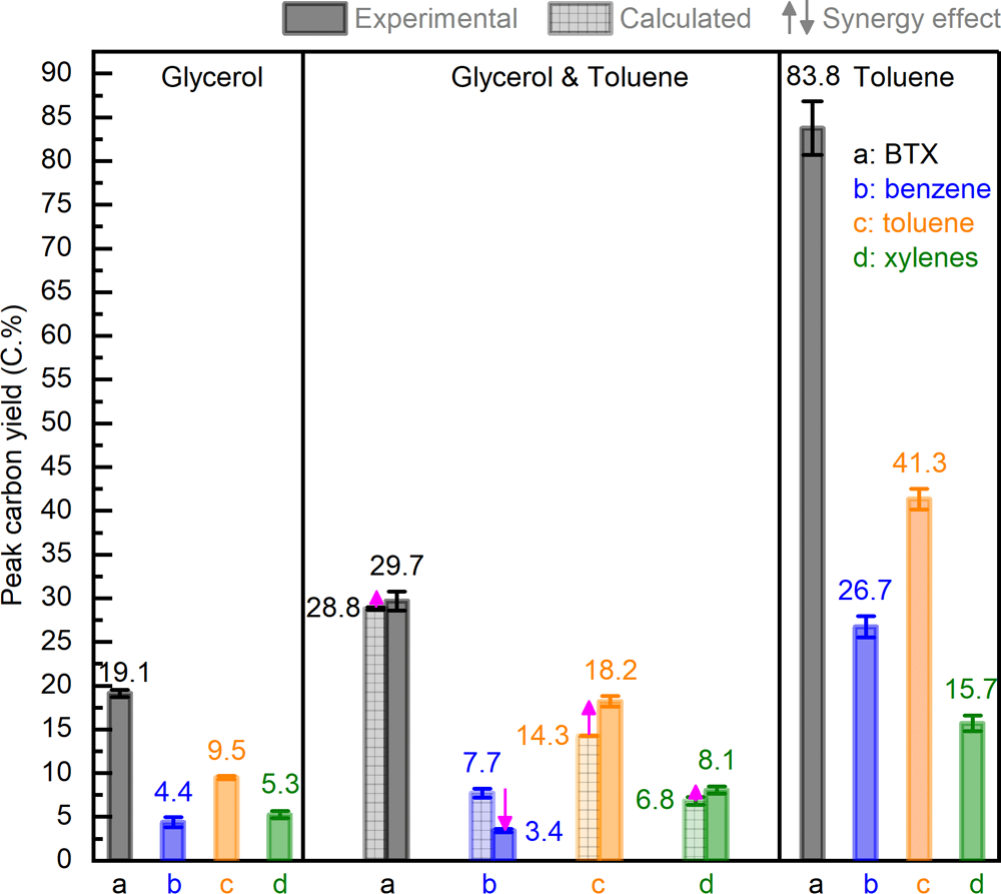
Peak carbon yield of BTX and individual benzene, toluene, and xylenes for catalytic conversion of glycerol (left) and toluene (right) and coconversion of glycerol and toluene (93/7 wt %, middle). The average value and standard deviation are calculated using the carbon yields at TOS of 1.5, 2, and 2.5 h. Reaction conditions: H-ZSM-5/Al_2_O_3_ (60/40 wt %) catalyst of 10 g, WHSV of the (co)feeds of 1 h^–1^, N_2_ flow of 50 mL min^–1^, reactor temperature of 550 °C, and atmospheric pressure.

**Figure 4:**
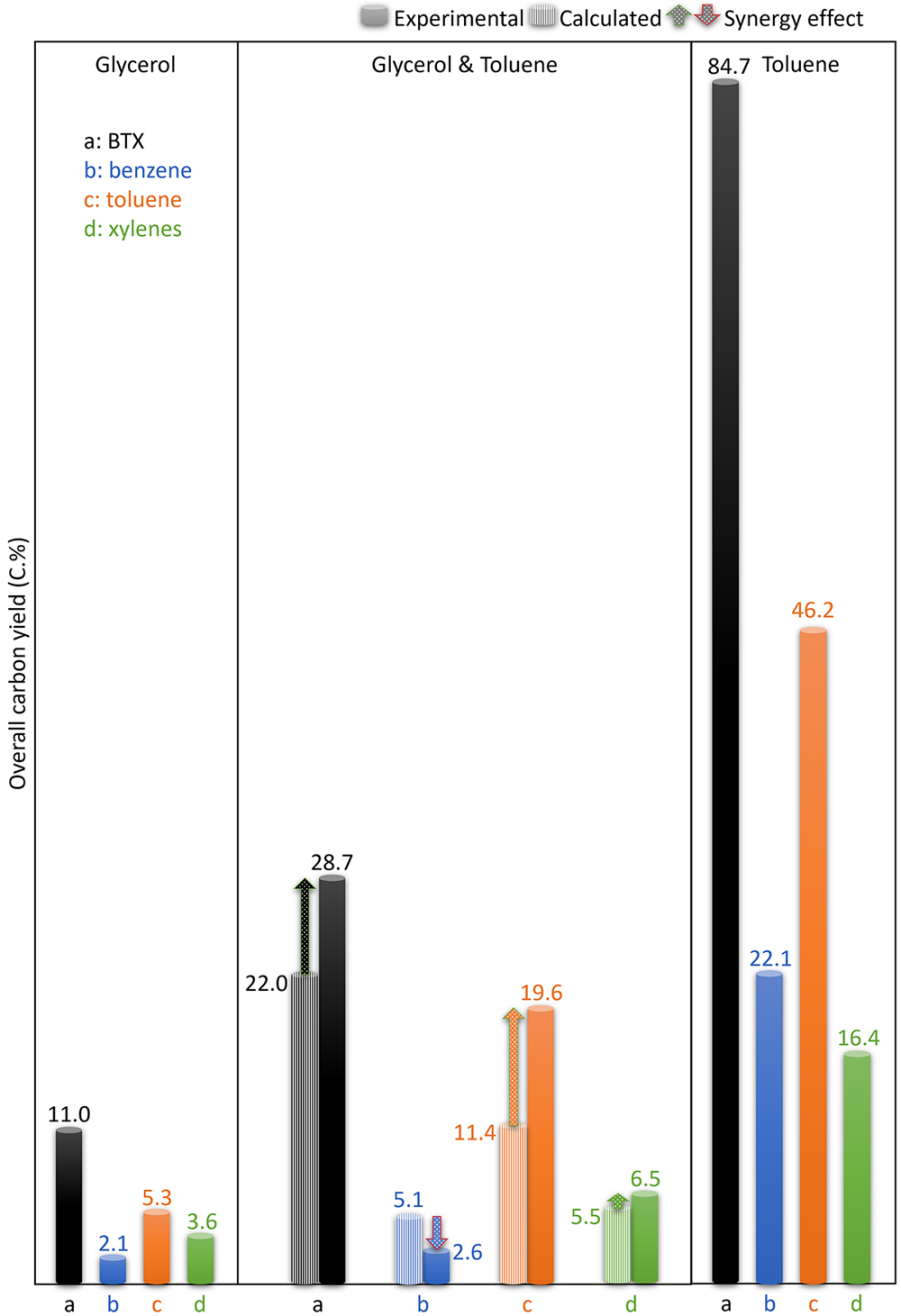
Overall carbon yield of BTX and individual benzene, toluene, and xylenes for catalytic conversion of glycerol (left) and toluene (right) and coconversion of glycerol and toluene (93/7 wt %, middle). Reaction conditions: H-ZSM-5/Al_2_O_3_ (60/40 wt %) catalyst of 10 g, WHSV of the (co)feeds of 1 h^–1^, N_2_ flow of 50 mL min^–1^, reactor temperature of 550 °C, atmospheric pressure, and TOS of 8.5 h.

The aromatic carbon yields for the catalytic conversion of glycerol over the H-ZSM-5/Al_2_O_3_ (60/40 wt %) catalyst are shown in Figure 2-a.^29^ The experiments were duplicated, and the average values including the corresponding errors are reported, showing a good repeatability of the experiments. The yield of aromatics increased rapidly in the initial TOS of 0.5–1.5 h, which is the so-called induction period often observed for GTA over H-ZSM-5-based catalysts,^26,33^ and is likely related to the time required to reach steady-state in the continuous setup.^27^ Afterward, the BTX carbon yield reached a maximum value of 19.1 ± 0.4 C.% at TOS of 1.5–2.5 h (Figure 2-a), which is termed as peak BTX carbon yield (Figure 3-left). The catalyst deactivated gradually with TOS, and the BTX carbon yields were below 1 C.% after a TOS of 8.5 h (Figure 2-a). The latter is defined as the catalyst lifetime. During the first run of the fresh catalyst for TOS of 8.5 h and without catalyst regeneration, the overall BTX carbon yield was 11.0 C.% (on feed basis, Figure 4-left), and the BTX production was 0.426 ton ton^–1^_catalyst_ (Figure S3-left, on catalyst basis, equivalent to 0.710 ton ton^–1^_ZSM-5_). The latter is defined as the BTX productivity on the catalyst during the first run. This benchmark performance represents the best results for pure glycerol conversion to BTX aromatics.^41^

### Catalytic Conversion of Toluene

Carbon yields of aromatics for the catalytic conversion of toluene over the H-ZSM-5/Al_2_O_3_ (60/40 wt %) catalyst are shown in Figure 2-c. This reaction is well-known as toluene disproportionation and is an important industrial process to produce benzene and xylenes from toluene.^42^ It is worth mentioning that there was no intention in this study to optimize the performance of the toluene disproportionation reaction. However, a very high toluene conversion of 60.0 ± 1.8 C.% was observed using the H-ZSM-5/Al_2_O_3_ (60/40 wt %) catalyst during the first 2 h TOS (a relatively stable period), yielding 41.7 ± 1.9 C.% of benzene and xylenes (Figure 2-c), 3.3 ± 0.3 C.% of other aromatics (Figure 2-c, including ethylbenzene, naphthalene, 1- and 2-methyl naphthalene), 1.0 ± 0.2 C.% of gaseous low-molecular-weight hydrocarbons (Figure S4-c, including CH_4_, C_2_H_6_, C_2_H_4_, C_3_H_8_, and C_3_H_6_), and others not collected (Figure S5-c, giving an overall mass balance closure of 89.3%). All yields are on a feed basis. Without taking catalyst regenerability into consideration, these performance data are comparable with those reported for commercial catalysts (toluene conversion of 30–50% and benzene and xylenes selectivity of 75.8–92.7%)^43^ and recently developed catalysts.^42,44,45^ Nevertheless, the selectivity of xylenes is lower compared to that of benzene (Figure S6-c), indicating the involvement of dealkylation reactions.^43^

### Catalytic Coconversion of Glycerol/Toluene

When toluene was cofed with glycerol, at a relatively low mass ratio of 93/7 wt %, the BTX carbon yield increased considerably compared to the catalytic conversion of glycerol only (Figure 2-b vs -a). The peak BTX carbon yield reached 29.7 ± 1.1 C.% (Figure 3-middle) at TOS of 1.5–2.5 h, which is by far higher than that for the catalytic conversion of glycerol alone (19.1 ± 0.4 C.%, Figure 3-left) and also for the catalytic coconversion of glycerol with various cofeeds including methanol, ethanol, dodecane, hexadecane, and oleic acid (21.7–26.7 C.%, in our recent work).^36^ Besides, the catalyst lifetime was prolonged to *ca*. 9–10 h (Figure 2-b). The productivity of BTX (including the toluene fraction for the recycle in cofeed, Figure S8-b) on the catalyst during the first run increased significantly with TOS. However, when the recycled toluene fraction is excluded, the “actual” BTX productivity on the catalyst during the first run is relatively stable (0.569 ± 0.012 ton ton^–1^_catalyst_, Figure S8-c) during the TOS of 8.5–12 h. This indicates negligible conversion of the cofeed at the prolonged TOS after 8.5 h, leading to a decreased overall BTX carbon yield (Figure S8-a). Therefore, the recommended TOS for the coconversion of glycerol and toluene (93/7 wt %) is 8.5 h, during which the overall BTX carbon yield was 28.7 C.% (on cofeed basis, Figure 4-middle), and the BTX productivity on the catalyst during the first run was 1.142 ton ton^–1^_catalyst_ (Figure S3-right, on catalyst basis, equivalent to 1.903 ton ton^–1^_ZSM-5_). This is the highest BTX productivity on the catalyst (per cycle of reaction-regeneration) reported to date for the catalytic (co)conversion of glycerol using ZSM-5-based catalysts.^41^ Nevertheless, the once-through catalyst consumption is *ca*. 876 kg_catalyst_ ton^–1^_BTX_, which is too high according to the established catalysis performance metrics.^40^ This means that the catalyst needs to be recycled a minimum of 876 times to meet the industrial requirements, which is difficult to implement practically in a fixed-bed reactor. One of the strategies might be to apply a cyclic/continuous catalyst regeneration (CCR) or fluid catalytic cracking (FCC) type of reactor to perform the reaction-regeneration cycles.^27^ The methodology of removing a small portion of the complete inventory of the regenerator and replacing it with the fresh catalyst to compensate for rapid catalyst deactivation, which has been practically successful in FCC process,^46^ could be applied here as well. A laboratory-scale cofluid catalytic cracking unit,^47^ which consists of multiple feeding system for cofeeding various feedstocks (*e.g*., glycerol and toluene) and a circulation system for catalytic cracking in a downer and catalyst regeneration in a riser, has been designed and constructed. The relevant research on catalyst reaction-regeneration cycles is under investigation and will be reported in due course.

### Synergistic Effect for Glycerol/Toluene

To identify whether cofeeding of toluene leads to synergetic effects, the theoretical catalyst performance data including peak and overall carbon yields of total and individual BTX aromatics were calculated based on the feed ratio of the individual feeds (85/15 on a carbon basis, equivalent to 93/7 on a mass basis) and the experimental data (shown in Figure 2-a,c). The calculation procedure has been described in detail in our previous paper^36^ and is included in the Supporting Information. The calculated values for the peak BTX carbon yield (28.8 ± 0.2 C.% at TOS of 1.5–2.5 h, Figure 3-middle), the overall BTX carbon yield (22.0 C.% during TOS of 8.5 h, Figure 4-middle), and the BTX productivity on the catalyst during the first run (0.900 ton ton^–1^_catalyst_ during TOS of 8.5 h, Figure S3-right) for the catalytic coconversion of glycerol/toluene (93/7 wt %) are all smaller compared to the experimental data, indicating a synergistic effect when cofeeding toluene. The quantified synergetic effects for the coconversion of glycerol and toluene (93/7 wt %) are expressed as a relative increase of 3.1% for the peak BTX carbon yield at TOS of 1.5–2.5 h and 30.0% for the overall BTX carbon yield during the TOS of 8.5 h. This synergistic effect has also been observed and quantified for the catalytic coconversion of glycerol with various other cofeeds such as alcohols, alkanes, and fatty acids. However, a sound explanation for the mechanisms behind this effect is still lacking.^36^ The slightly increased hydrogen-to-carbon effective ratio (H/C_eff_) of the cofeed glycerol/toluene compared to that of the individual feed glycerol (0.74 vs 0.67) is not expected to play such a significant role.^48^ Another explanation may be found when considering the gas-phase composition. The carbon yields of CO_2_ (related to decarboxylation) and, in particular, CO (attributed to decarbonylation) are dramatically increased upon cofeeding toluene with glycerol (Figure S7-b vs -a). This leads to intermediates with an increased H/C_eff_, which could subsequently be more effective in aromatization reactions in the so-called “hydrocarbon pool”.^32,49^ On the other hand, the selectivity of xylenes for the catalytic coconversion of glycerol/toluene (Figure S6-b) is higher than that of benzene, while those for the catalytic conversion of glycerol alone (Figure S6-a) and toluene (Figure S6-c) are lower. This indicates that alkylation reactions are most likely involved, may play a role in the mechanism, and may provide synergistic effects. This hypothesis is supported by the experimental data showing that the peak carbon yield (Figure 3-middle), overall carbon yield (Figure 4-middle), and productivity on the catalyst during the first run (Figure S3-right) of benzene are lower than the calculated values. This contrasts with the lower values for the calculated values of toluene and xylenes. Catalytic conversion of glycerol (alone) results in an intermediate hydrocarbon pool that is further converted to a mixture of aromatics, including BTX. The aromatics formed are sensitive to alkylation reactions originating from, *e.g*., the hydrocarbon pool. In the case of catalytic coconversion of glycerol and toluene, the alkyl groups, which are transferred among different aromatic rings during toluene disproportionation, might participate in the aromatization and alkylation reactions of intermediates from glycerol conversion. Very likely such interactions facilitate the synergistic effect; however, other explanations may not be ruled out. Nevertheless, detailed mechanistic studies, *e.g*., labeling studies using ^13^C- and/or D-labeled glycerol as the feed combined with advanced product analysis (*e.g*., using a GC-Orbitrap MS), will be required for a better understanding of the synergistic effects observed here.

### Self-Sufficient Toluene Product Recycling Strategy

**Figure 5:**
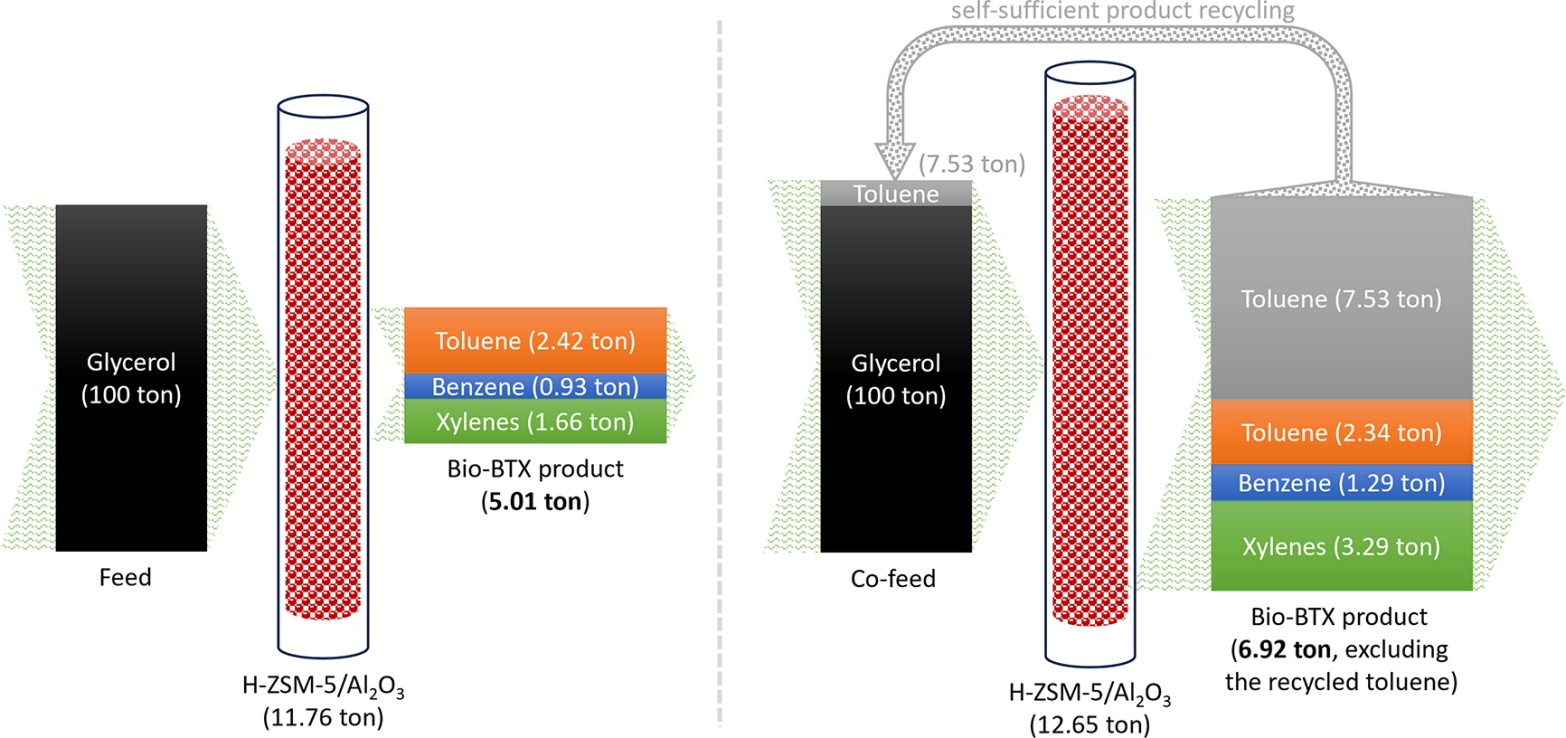
Enhanced production of bio-BTX aromatics (in particular, xylenes) for catalytic conversion of glycerol (left) by self-sufficient toluene product recycling (right). Reaction conditions: H-ZSM-5/Al_2_O_3_ (60/40 wt %), WHSV of the (co)feeds of 1 h^–1^, N_2_ flow of 50 mL min^–1^, reactor temperature of 550 °C, atmospheric pressure, and TOS of 8.5 h.

With these findings above, the cofeeding strategy using the *in situ* produced toluene product as the cofeed to enhance the production of bio-BTX aromatics from glycerol is proposed in Figure 5, showing that the *in situ* produced toluene is self-sufficient for recycling. As illustrated in Figure S9, for a catalytic coconversion of glycerol/toluene process using 1 ton of H-ZSM-5/Al_2_O_3_ catalyst, 8.5 tons of cofeed (containing 7.905 tons of glycerol and 0.595 tons of toluene with a mass ratio of 93/7) can be converted to 0.102 tons of benzene, 0.780 tons of toluene, and 0.260 tons of xylenes. The recycled toluene in the cofeed is 76% of the toluene in the product. Considering that this fraction of toluene needs to be recycled in cofeed, the “actual” BTX productivity on the catalyst during the first run for catalytic coconversion of glycerol/toluene is 0.547 ton ton^–1^_catalyst_ (Figure S9). This sustainable catalysis performance is *ca*. 28% higher than that for catalytic conversion of glycerol (0.426 ton ton^–1^_catalyst_, Figure S9). The present cofeeding strategy is, in particular, beneficial for producing the biobased xylenes with a relative increase of 84.4% in productivity on the catalyst during the first run and 43.5% in selectivity (Figure S9). Considering favorable benzene alkylation and toluene disproportionation, the increase in the selectivity of xylenes may be more significant when using higher toluene/glycerol ratios (up to a certain point) in the cofeed. However, the cofeed ratio needs to be varied to fully understand its effect on BTX productivity on the catalyst during the first run and selectivity, and this has not yet been investigated in detail.

Herewith, a novel self-sufficient product recycling strategy for a remarkably improved GTA performance, including BTX yield (on the feed basis), BTX productivity (on the catalyst basis and during the first run), and xylenes’ selectivity (on the BTX product basis), has been proposed. In this proof of concept: (i) Biobased toluene product is recycled directly (without additional catalytic conversion) as cofeed. This is very attractive for the simplification of the product recycling process. (ii) A portion of the *in situ* produced toluene is recycled as cofeed, and the remainder is collected as the BTX composition to improve and stabilize BTX production. This is of great significance to improve the glycerol conversion efficiency. (iii) The selectivity and production of xylenes are dramatically increased. This is particularly interesting considering bioxylenes are the most desired bioaromatics. To further demonstrate this self-sufficient product recycling strategy and understand the mechanism of the enhanced GTA performance, catalytic copyrolysis of glycerol with the other two BTX products (namely, benzene and xylenes) and the oxygenated products (Figure 1, such as acetaldehyde and acrolein^26^) using advanced isotopic labeling and detailed catalyst reaction-regeneration cycles are of interest and will be the subject of future studies. This self-sufficient product recycling strategy significantly increases the usage efficiency of green carbon from biomass to the desired products, enhancing environmental and economic sustainability. Therefore, a detailed technoeconomic and life-cycle assessment is another interesting subject of future studies.

## Data Availability

All data are available in the main text or the Supporting Information.

## Supporting Information Available

Extended experimental procedure and product analysis; Pricing for BTX (Figure S1); synthesis of polymers from benzene and xylenes (Figure S2); BTX productivity on the catalyst during the first run (Figure S3); carbon yields of low-molecular-weight hydrocarbons (Figure S4); mass balance (Figure S5); BTX selectivity (Figure S6); carbon yields of CO and CO_2_ (Figure S7) for catalytic conversion of individual glycerol and toluene and catalytic coconversion of glycerol and toluene; overall BTX carbon yield and productivity of BTX on the catalyst during the first run for catalytic coconversion of glycerol and toluene at varied TOS (Figure S8); the scheme of self-sufficient toluene product recycling stretagy for the enhanced production of bio-BTX aromatics (in particular xylenes) for catalytic conversion of glycerol (Figure S9); and additional references (PDF)
