# Supplementary material for: Enhanced Bio-BTX Formation by Catalytic Pyrolysis of Glycerol with In Situ Produced Toluene as the Cofeed
Source: ACS Sustain Chem Eng. 2024 Mar 25;12(15):5731–7. doi: 10.1021/acssuschemeng.4c00451 (PMC11022236; doi:10.1021/acssuschemeng.4c00451)
Supplement: Supplementary file 1 — sc4c00451_si_001.pdf [file sc4c00451_si_001.pdf]

Supporting Information

Enhanced bio-BTX formation by catalytic pyrolysis of glycerol with *in-situ* produced toluene as the co-feed

Fukang Wang,[a,#] Thomas Sjouke Kramer,[b,#] Bin Yan,[a] Lin Zhu,[a] Yuezhao Zhu,[a] Andre Heeres,[c] Diana Ciolca,[d] Hero Jan Heeres,[b,*] and Songbo He[a,*]

[a]Joint International Research Laboratory of Circular Carbon, Nanjing Tech University, Nanjing 211816, PR China

[b]Green Chemical Reaction Engineering, Engineering and Technology Institute Groningen, University of Groningen, 9747 AG Groningen, The Netherlands

[c]Hanze University of Applied Sciences, 9747 AS Groningen, The Netherlands

[d]BioBTX BV, 9747 AA Groningen, The Netherlands

*Corresponding authors. E-mail: h.j.heeres@rug.nl (H.J. Heeres) and songbo.he@njtech.edu.cn (S. He)

Number of pages: 15

Number of figures: 9

Contents

Experimental………..S3

Calculation procedure for the calculated data shown in Figs. 3, 4, S3, and S4…………………...S5

Figure S1. Pricing for benzene, toluene, mixed xylenes, and crude oil in 2017 - 2023. ……….S6

Figure S2. Synthesis of bio-based polymers from bio-based benzene and xylenes. ……………S7

Figure S3. Productivity of (a) total BTX, (b) benzene, (c) toluene, and (d) xylenes on the catalyst during the first run for catalytic conversion of glycerol (left) and co-conversion of glycerol and toluene (93/7 wt.%, right). ……………………………………………………………………………………………S8

Figure S4. Carbon yields of low-molecular-weight hydrocarbons *versus* TOS for catalytic conversion of individual glycerol (a) and toluene (c), and catalytic co-conversion of glycerol and toluene (93/7 wt.%, b). ……………………………………………………………………………………...….S9

Figure S5. Mass balance for catalytic conversion of individual glycerol (a) and toluene (c), and catalytic co-conversion of glycerol and toluene (93/7 wt.%, b)………………………………….……..S10

Figure S6. Carbon selectivity of the individual BTX in total BTX *versus* TOS for catalytic conversion of individual glycerol (a) and toluene (c), and catalytic co-conversion of glycerol and toluene (93/7 wt.%, b). ………………………………………………………………………………….……..S11

Figure S7. Carbon yields of CO and CO_2 *versus* TOS for catalytic conversion of individual glycerol (a) and toluene (c), and catalytic co-conversion of glycerol and toluene (93/7 wt.%, b). ……..S12

Figure S8. Overall BTX carbon yield (a), productivity of BTX on the catalyst during the first run including the toluen fraction for the recycle in co-feed (b), and productivity of BTX on the catalyst during the first run excluding the toluen fraction recycled in co-feed (c) for co-conversion of glycerol and toluene (93/7 wt.%). ……………………………………………………………S13

Figure S9. Enhanced production of bio-BTX aromatics (in particular xylenes) for catalytic conversion of glycerol (left) by self-sufficient toluene product recycling (right). ………………..S14

References ………S15

Experimental

Materials. A granular alumina bonded H-ZSM-5 catalyst (H-ZSM-5/Al_2O_3, 60/40 wt.%, ϕ 1.2 - 1.8 mm, SiO_2/Al_2O_3 molar ratio of 28) was produced at Yangzhou Baisheng Catalyst Co., Ltd., PR China. The as-received H-ZSM-5/Al_2O_3 catalyst was calcined at 600 °C 8 h and the calcined catalyst was stored in a vacuum desiccator. Glycerol (> 99.5% purity) was supplied by Boom BV, The Netherlands. Toluene, ethanol, tetrahydrofuran (THF), and n-nonane were of analytical grade and supplied by Sigma-Aldrich. The liquid and gaseous N_2 were supplied by Linde.

Catalytic (co-)pyrolysis experiemnts. The experiments were performed on a well-engineered bench-scale fixed bed reactor setup. The fresh H-ZSM-5/Al_2O_3 catalyst (10 g) was loaded to the isothermal zone of a stainless steel tubular reactor, of which the rest was filled with quartz wool. The reactor was heated to 550 °C under an N_2 flow of 50 ml min^{-1}. The (co-)feed was pumped (10 g h^{-1}) into a preheater (maintained at 350 °C) by using the syringe pump(s) and the gastight syringe(s). The vapor mixed with N_2 gas was first passed to the vent to purge the system for *ca*. 2 h and then introduced to the catalyst reactor. The reactions were continuously performed for a TOS of 12 h. Liquid samples (collected in a 20-ml glass vial) and gas-phase samples (collected in a 5-L FlexFoil Plus sample bag) were taken every 30 min.

Product analysis. The liquid products collected in the 20-ml glass vials were diluted approximately 7 times with a stock solution (*ca*. 20,000 ppm of *n*-nonane in a mixed solvent containing THF and ethanol with a volume ratio of *ca*. 1 to 10) to prepare homogeneous samples for various off-line analyses. The mixtures were analyzed by GC-MS (6890/5973, Hewlett-Packard) and GC-FID (5890, Hewlett-Packard) equipped with a Rtx-1701 column (30 m × 0.25 mm × 0.25 µm, Restek). The relative response factors of the individual aromatics to the internal standard, *viz*., *n*-nonane, were applied for the quantification of aromatics. The carbon, hydrogen, and oxygen contents in the samples were analyzed using a EuroEA3000 elemental analyzer (Eurovector). The gaseous products collected in the 5-L gas bags were analyzed by GC-TCD (5890, Hewlett-Packard) equipped with a CP-PoraBOND Q column (50 m × 0.53 mm × 10 µm, Varian) and an HP-Molsieve column (30m x 0.53 mm x 50 µm, Agilent). Before and after the analyses, the GC-TCD was pre- and post-calibrated with a standard reference gas mixture containing C_1-C_3, CO, CO_2, and N_2. The carbon yields of products, the

selectivity of individual BTX components, and BTX productivity on the catalyst during the first run were calculated using **Equations 1-3**.

$$\text{Yield (\%, on carbon basis)} = \frac{\text{mol of carbon in the individual product}}{\text{mol of carbon in the feed}} \times 100 \quad (1)$$

$$\text{BTX selectivity (\%)} = \frac{\text{mol of individual BTX component produced}}{\text{mol of total BTX product}} \times 100 \quad (2)$$

$$\text{BTX productivity } \left(mg_{BTX}\, g_{catalyst}^{-1}\right) = \frac{\text{weight of BTX produced}}{\text{weight of catalyst loaded in the reactor}} \quad (3)$$

Calculation procedure for the calculated data shown in Figs. 3, 4, S3, and S4

The following general calculation equation was used:

Calculated carbon yield of the co-feed = Experimental performance data of Feed A × carbon fraction (C.%) of Feed A in the co-feed + Experimental performance data of Feed B × carbon fraction (C.%) of Feed B in the co-feed

The mass ratio of glycerol and toluene in the co-feed was 93/7 wt.%.

The carbon ration of glycerol and toluene in the co-feed was 85/15 C.%.

Calculated data in Figs. 3 and S3

	Glycerol (Experimental)	Toluene (Experimental)	Glycerol/Toluene (85/15 C.%) (Calculated)
Carbon yield of benzene (C.%)#	4.4	26.7	4.4×0.85+26.7×0.15=7.7
Carbon yield of toluene (C.%) #	9.5	41.3	9.5×0.85+41.3×0.15=14.3
Carbon yield of xylenes (C.%) #	5.3	15.7	5.3×0.85+15.7×0.15=6.8

#The average value of the carbon yields at TOS of 1.5, 2, and 2.5 h.

Calculated data in Fig. 4

	Glycerol (Experimental)	Toluene (Experimental)	Glycerol/Toluene (85/15 C.%) (Calculated)
Overall carbon yield of benzene (C.%)	2.1	22.1	2.1×0.85+22.1×0.15=5.1
Overall carbon yield of toluene (C.%)	5.3	46.2	5.3×0.85+46.2×0.15=11.4
Overall carbon yield of xylenes (C.%)	3.6	16.4	3.6×0.85+16.4×0.15=5.5

Calculated data in Fig. S4

	Glycerol (Experimental)	Toluene (Experimental)	Glycerol/Toluene (93/7 wt.%) (Calculated)
Productivity of benzene on the catalyst during the first run (mg $g^{-1}_{catalyst}$)	79	1859	79×0.93+1859×0.07=204
Productivity of toluene on the catalyst during the first run (mg $g^{-1}_{catalyst}$)	206	3926	206×0.93+3926×0.07=466
Productivity of xylenes on the catalyst during the first run (mg $g^{-1}_{catalyst}$)	141	1409	141×0.93+1409×0.07=230

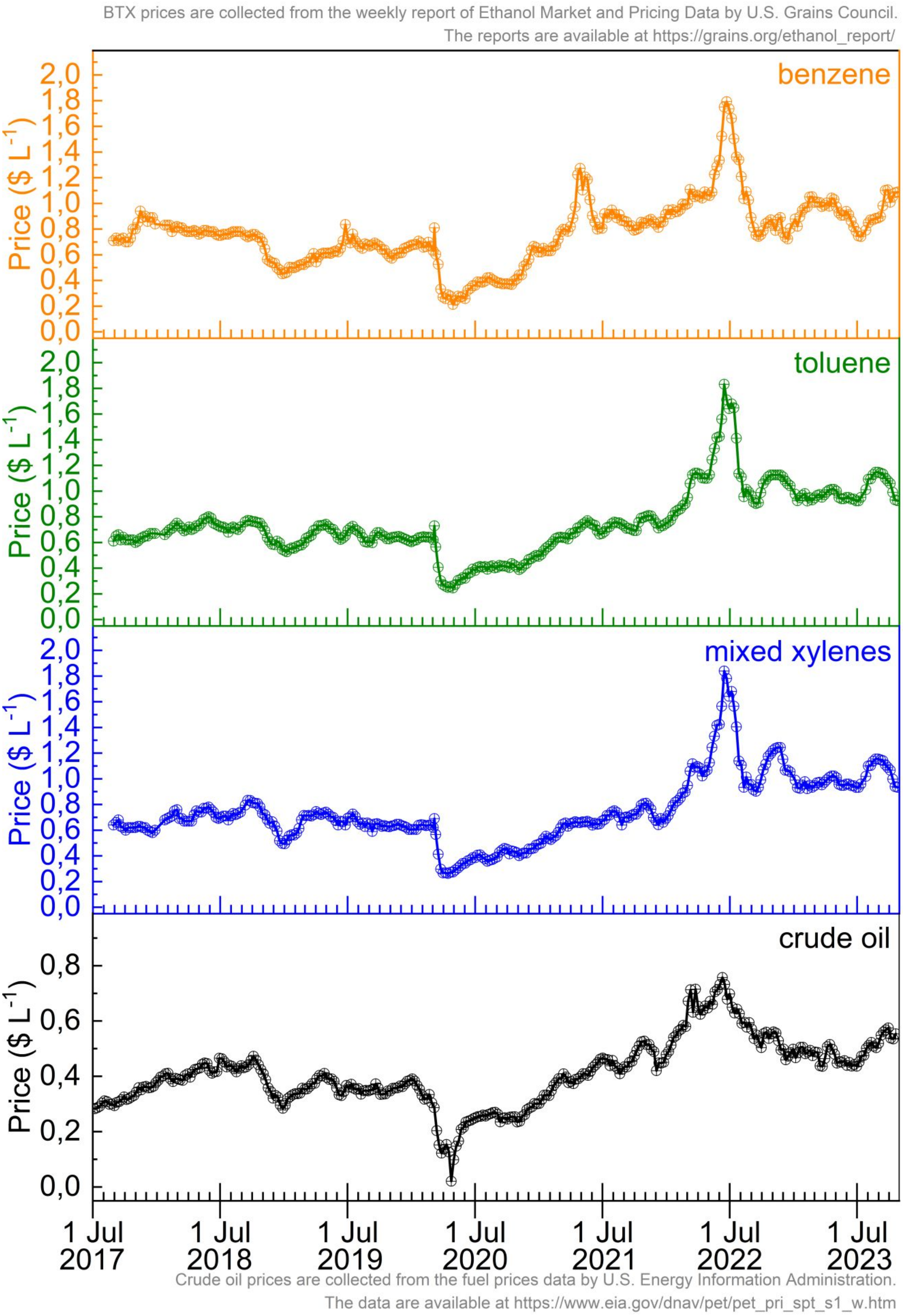

Figure S1. Pricing for benzene, toluene, mixed xylenes, and crude oil in 2017 - 2023.[S1-S2]

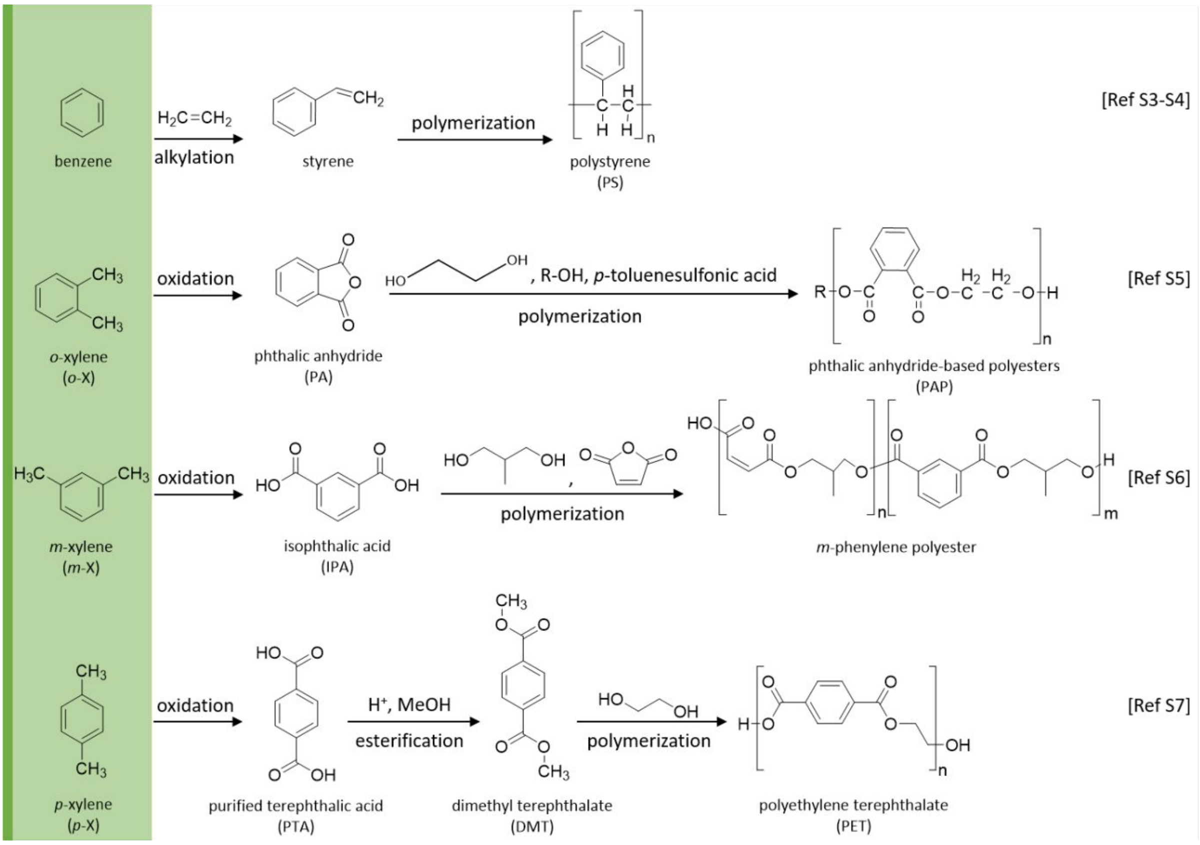

Figure S2. Synthesis of bio-based polymers from bio-based benzene and xylenes.[S3-S7]

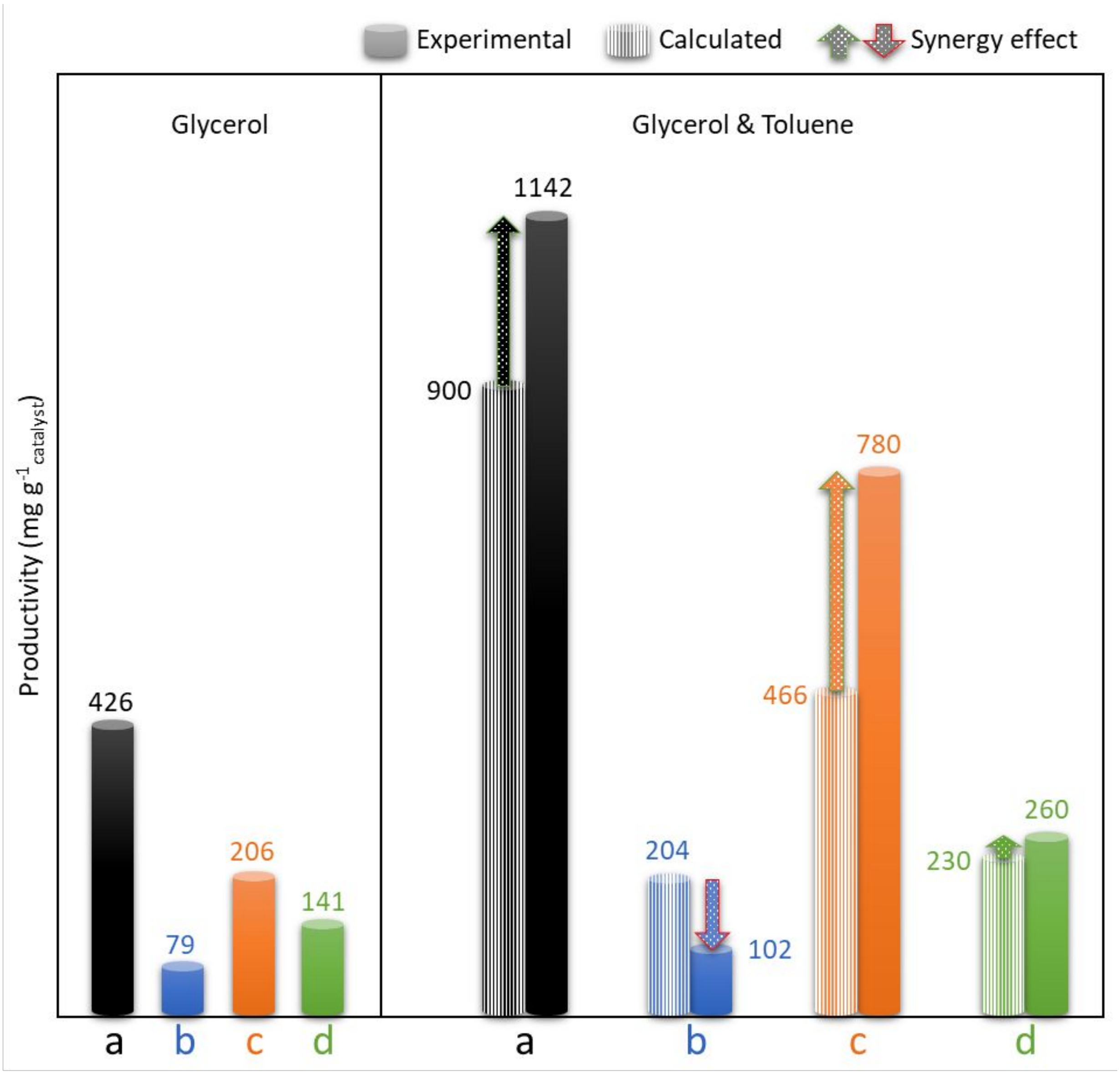

Figure S3. Productivity of (a) total BTX, (b) benzene, (c) toluene, and (d) xylenes on the catalyst during the first run for catalytic conversion of glycerol (left) and co-conversion of glycerol and toluene (93/7 wt.%, right). Reaction conditions: H-ZSM-5/Al_2O_3 (60/40 wt.%) catalyst of 10 g, WHSV of the (co-)feeds of 1 h^{-1}, N_2 flow of 50 ml min^{-1}, reactor temperature of 550 °C, atmospheric pressure, and TOS of 8.5 h.

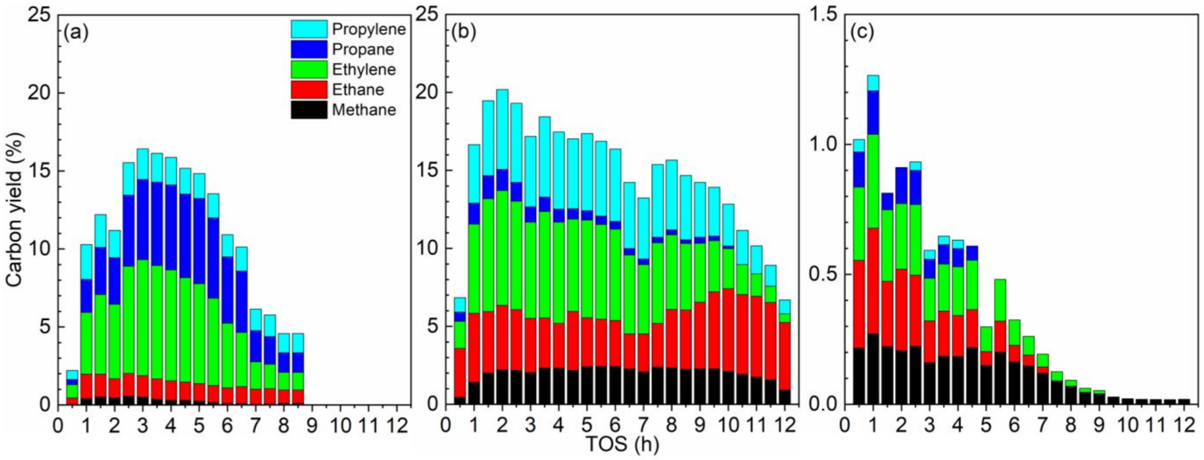

Figure S4. Carbon yields of low-molecular-weight hydrocarbons *versus* TOS for catalytic conversion of individual glycerol (a) and toluene (c), and catalytic co-conversion of glycerol and toluene (93/7 wt.%, b). Reaction conditions: H-ZSM-5/Al_2O_3 (60/40 wt.%) catalyst of 10 g, WHSV of the (co-)feeds of 1 h^{-1}, N_2 flow of 50 ml min^{-1}, reactor temperature of 550 °C, and atmospheric pressure.

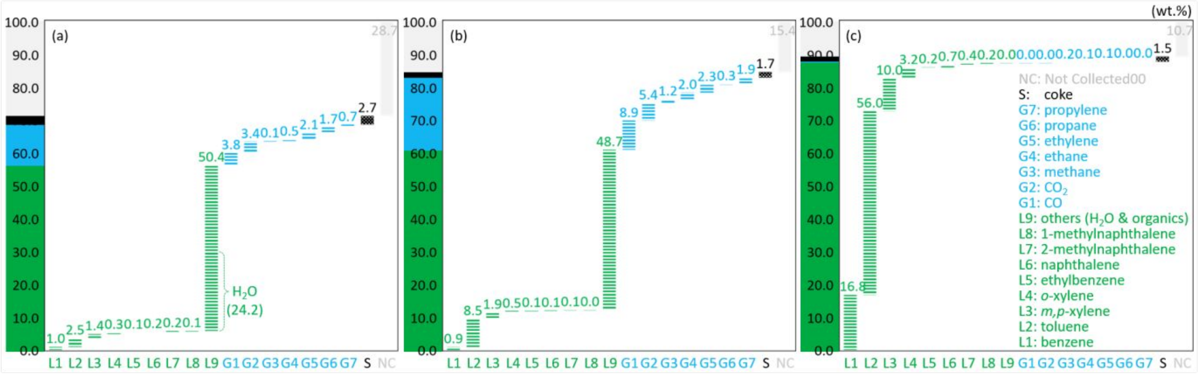

Figure S5. Mass balance for catalytic conversion of individual glycerol (a) and toluene (c), and catalytic co-conversion of glycerol and toluene (93/7 wt.%, b). Reaction conditions: H-ZSM-5/Al_2O_3 (60/40 wt.%) catalyst of 10 g, WHSV of the (co-)feeds of 1 h^{-1}, N_2 flow of 50 ml min^{-1}, reactor temperature of 550 °C, and atmospheric pressure.

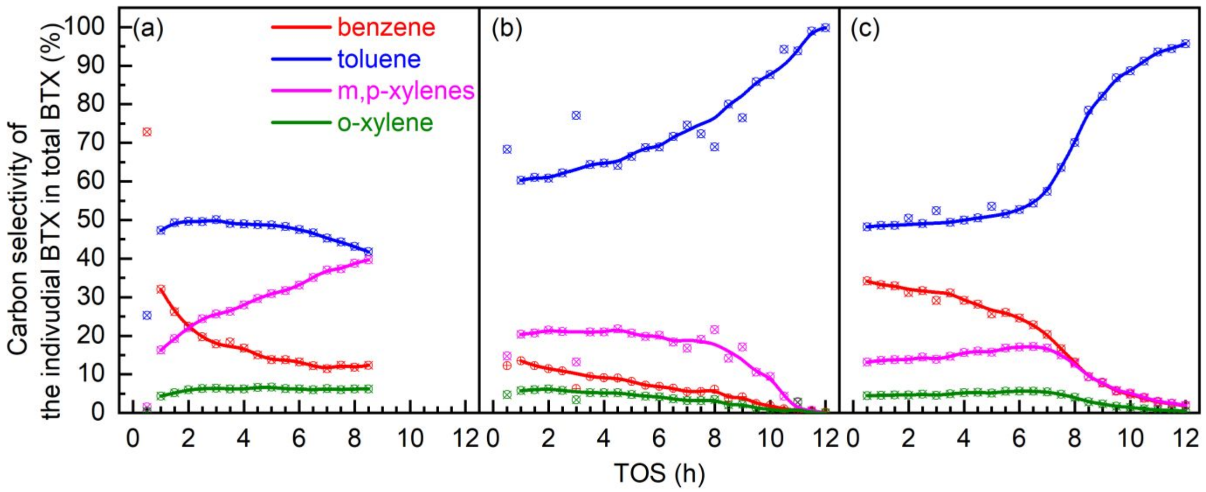

Figure S6. Carbon selectivity of the individual BTX in total BTX *versus* TOS for catalytic conversion of individual glycerol (a) and toluene (c), and catalytic co-conversion of glycerol and toluene (93/7 wt.%, b). Reaction conditions: H-ZSM-5/Al_2O_3 (60/40 wt.%) catalyst of 10 g, WHSV of the (co-)feeds of 1 h^{-1}, N_2 flow of 50 ml min^{-1}, reactor temperature of 550 °C, and atmospheric pressure.

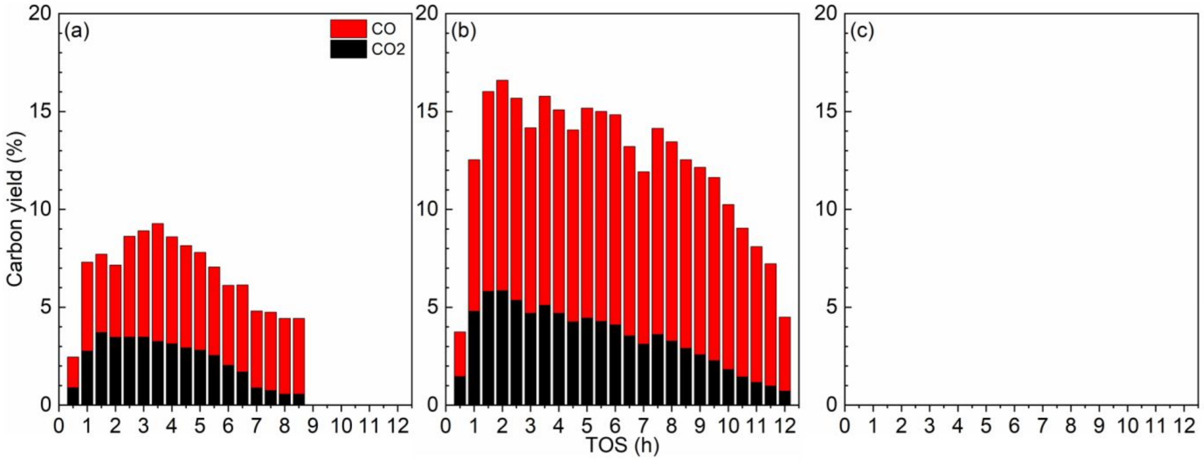

Figure S7. Carbon yields of CO and CO_2 *versus* TOS for catalytic conversion of individual glycerol (a) and toluene (c), and catalytic co-conversion of glycerol and toluene (93/7 wt.%, b).

Reaction conditions: H-ZSM-5/Al_2O_3 (60/40 wt.%) catalyst of 10 g, WHSV of the (co-)feeds of 1 h^{-1}, N_2 flow of 50 ml min^{-1}, reactor temperature of 550 °C, and atmospheric pressure.

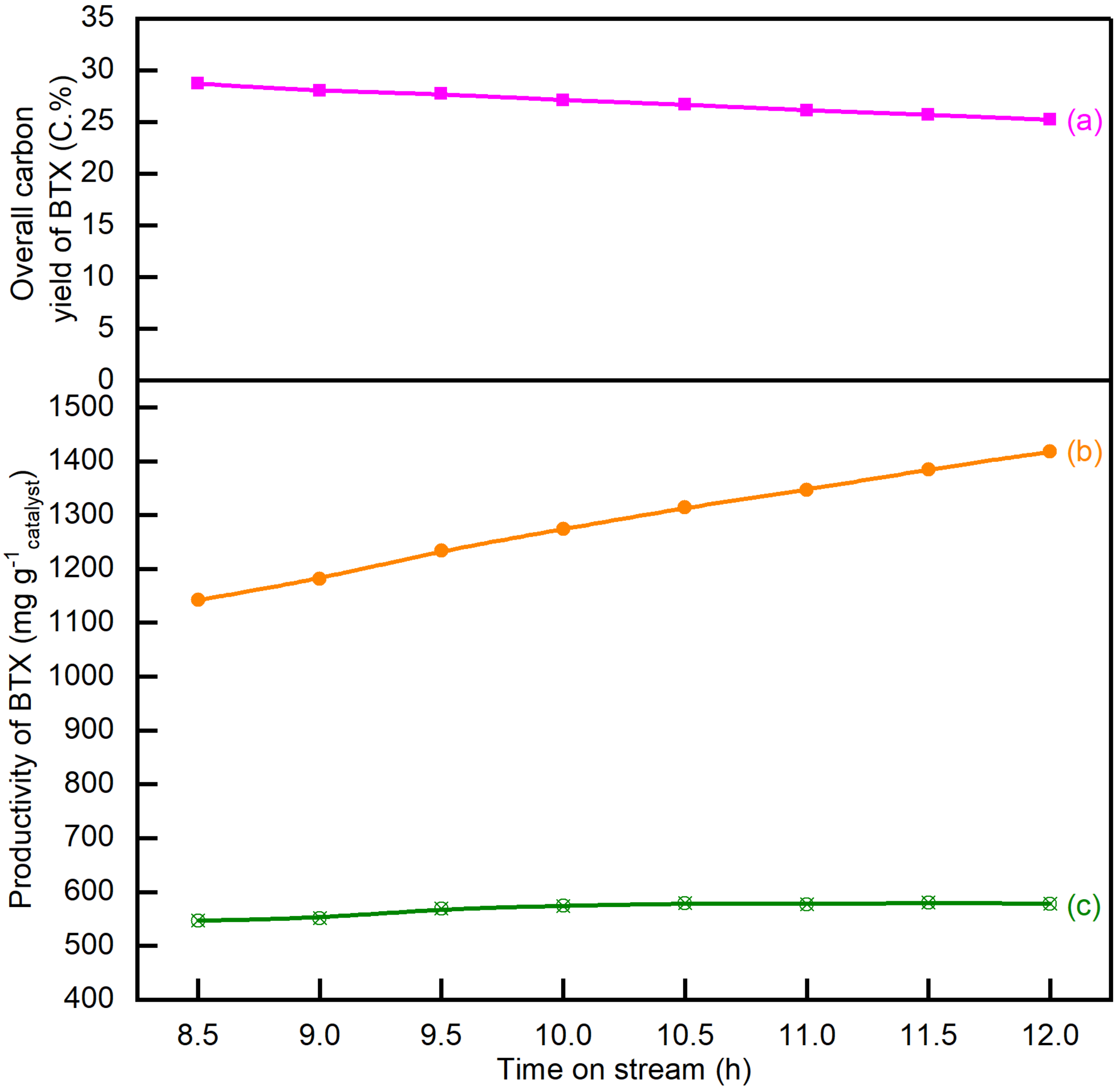

Figure S8. Overall BTX carbon yield (a), productivity of BTX on the catalyst during the first run including the toluene fraction for the recycle in co-feed (b), and productivity of BTX on the catalyst during the first run excluding the toluen fraction recycled in co-feed (c) for co-conversion of glycerol and toluene (93/7 wt.%). Reaction conditions: H-ZSM-5/Al_2O_3 (60/40 wt.%), WHSV of co-feed of 1 h^{-1}, N_2 flow of 50 ml min^{-1}, reactor temperature of 550 °C, atmospheric pressure, and TOS varied from 8.5 to 12 h.

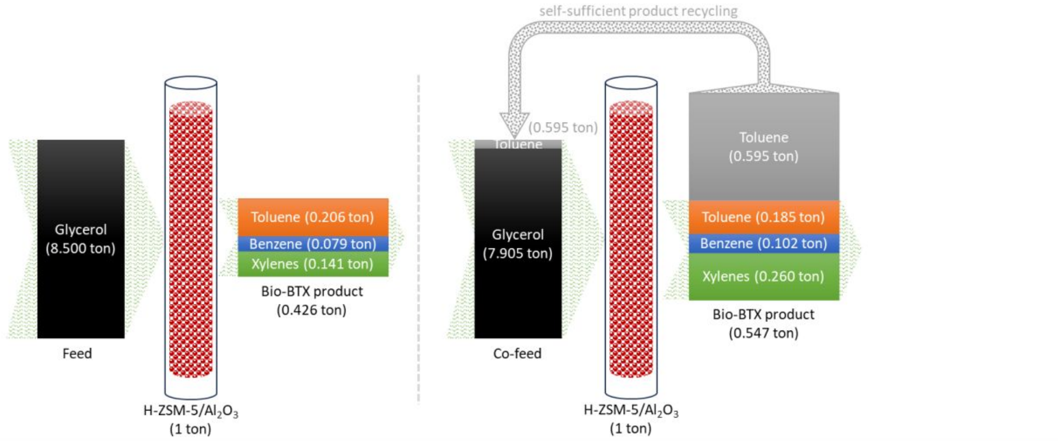

Figure S9. Enhanced production of bio-BTX aromatics (in particular xylenes) for catalytic conversion of glycerol (left) by self-sufficient toluene product recycling (right). Reaction conditions: H-ZSM-5/Al_2O_3 (60/40 wt.%), WHSV of the (co-)feeds of 1 h^{-1}, N_2 flow of 50 ml min^{-1}, reactor temperature of 550 °C, atmospheric pressure, and TOS of 8.5 h.

References

S1. Ethanol Market and Pricing Data, https://grains.org/ethanol_report/, (accessed 27th October, 2023).

S2. Petroleum & other liquids - Spot prices, https://www.eia.gov/dnav/pet/pet_pri_spt_s1_w.htm, (accessed 27th October, 2023).

S3. l-Shall, M. S.; Bahta, A.; Rabeony, H.; Reiss, H., Homogeneous gas phase thermal polymerization of styrene. *J. Chem. Phys.*, 1987, **87**, 1329-1345. (https://doi.org/10.1063/1.453315)

S4. Vaughan, B. A.; Webster-Gardiner, M. S.; Cundari, T. R.; Gunnoe, T. B., A rhodium catalyst for single-step styrene production from benzene and ethylene. *Science*, 2015, **348**, 421-424. (https://doi.org/doi:10.1126/science.aaa2260)

S5. Albu, P.; Vlase, G.; Modra, D.; Bolcu, C.; Vlase, T., Thermal behaviour of the polyesters obtained with different molar ratios of carboxyl hydroxyl components. *J. Therm. Anal. Calorim*., 2015, **121**, 1031-1037. (https://doi.org/10.1007/s10973-015-4783-x)

S6. Bartoli, M.; Rosi, L.; Frediani, M., Chapter 22 - Synthesis and Applications of Unsaturated Polyester Composites. In *Unsaturated Polyester Resins*, Thomas, S.; Hosur, M.; Chirayil, C. J., Eds. Elsevier: 2019; pp. 579-598. (https://doi.org/10.1016/B978-0-12-816129-6.00022-3)

S7. Hwang, K.-R.; Jeon, W.; Lee, S. Y.; Kim, M.-S.; Park, Y.-K., Sustainable bioplastics: Recent progress in the production of bio-building blocks for the bio-based next-generation polymer PEF. *Chem. Eng. J.*, 2020, **390**, 124636. (https://doi.org/https://doi.org/10.1016/j.cej.2020.124636)
